# Cases Showing the Frequency of the Occurrence of Follicular Ulceration in the Mucous Membrane of the Intestines, during the Progress of Idiopathic Fevers; with Dissections, and Observations on Its Pathology

**Published:** 1826-08

**Authors:** Cornwallis Hewett

**Affiliations:** Physician to St. George's Hospital.


THE LONDON

### Medical and Physical Journal.

No. 330, VOL. LVI.] AUGUST, 1826. [No. 2, *New Series*.

For many fortunate discoveries in medicine, and for the detection of numerous errors, the world is indebted to the rapid circulation of Monthly Journals; and there never existed any work, to which the Faculty, in Europe and America, were under deeper obligations, than to the Medical and Physical Journal of London, now forming a long, but an invaluable, series.—RUSH.

### CASES

### OBTAINED FROM PUBLIC INSTITUTIONS, AND OTHER AUTHENTIC SOURCES.

### FOLLICULAR ULCERATION.

*Cases showing the Frequency of the Occurrence of Follicular Ulceration in the Mucous Membrane of the Intestines, during the Progress of Idiopathic Fevers; with Dissections, and Observations on its Pathology.* By CORNWALLIS HEWETT, M.D. Physician to ST. GEORGE'S HOSPITAL.

I. *Follicular Ulceration of the Mucous Membrane of the Intestines, without any tenderness on forcible pressure of the abdomen.*

MARY HANCOCK, aged thirty-two; admitted April 23, 1825.

Was attacked this day fortnight with rigors, vomiting, &c. but was not confined to bed till Tuesday se'nnight; when she was bled in consequence of giddiness, which however was unattended by headache. She has since been treated by a distinguished physician, in whose house she resided. Face flushed; eyes natural; skin dry, but not of pungent heat; pulse 108, small and soft. Is now perfectly rational, but has some slight subsultus. Tongue yellow-brown, and dry. Abdomen full, *but there is no tenderness on pressure.* One copious stool this morning. Has slept well last night, having taken an opiate; but had no sleep for several nights previously.

Hydrar. Submuriat. gr. v.; Pulv. Jacobi (veri), gr. iij. fiat pil. statim sumenda. Post horas tres sumat haustum sequent.

Liq. Antim. Tartar. ʒ ss.; Sulphat. Magnes. ʒ i.; Aquæ distillat. ʒ xj.; Syrupi, ʒ i. M. Repet. 4ta quaque hora.

Lotio frigida capiti, et appl. Hirudines xij. fronti, vesperi; si redierit vertigo vel delirium.

April 24th.—Is stated to have slept well. Face less flushed. Continual, though slight subsultus. Pulse 100, rather small; tongue moist, apparently cleaning; bowels freely opened; skin dry, not pungent.

Hydrar. Submur., Pulv. Jacobi (veri), Extract. Pap. alb. āā gr. iij. fiant pil. duæ, h. s. sumendæ.

25th.—Bowels very much purged; tongue moist, brown towards the root; skin cool; cheeks have lately become flushed. Slept well; has less subsultus; pulse 108, very small.

26th.—Purgative acted eight times in the afternoon, and three times during the night: the evacuations watery, but of good bilious appearance. *Not the slightest tenderness of the abdomen.* Skin cool; tongue dry and brown; eyes rather injected. Is perfectly rational. She slept well during the first part of the night, but about twelve she became so much exhausted, that the apothecary gave her a little brandy and water, and administered an opiate glyster.

Mistur. Camphoræ, ℥ xj.; Carbon. Ammoniæ, gr. v.; Confect. Aromat., Syrupi Aurantii, āā ℥ ss. M. Sumatur tertia quaque hora.

Misturæ Cretæ, ℥ xj.; Spir. Myristicæ, ℥ i.; Tinct. Opii, gtt. viij. fiat haustus post singulas dejectiones liquidas repet. Omitt. alia.

27th.—Bowels quiet. Camphor draught rejected.

Infus. Cascarillæ, ℥ x.; Tr. Ejusdem, ℥ ij.; Spir. Ammoniæ arom. ℥ ss. M. 3tiis horis.

Vini Hispan. ℥ ij. 6tis horis.

Similar remedies were continued, and the symptoms went on without any important change till the 2d of May. *In each daily report, it is mentioned that the abdomen, though rather inflated, was not in any degree tender.*

May 2d.—Has vomited her medicines, along with some green fluid,—apparently vitiated bile. Two stools since yesterday, loose, but otherwise healthy. Pulse very frequent and feeble; skin quite cool. Said to have been noisy during the night, but is now quite rational. Some petechiæ have made their appearance about the back and abdomen.

Sulphat. Quinæ, gr. jss.; Extract. Anthemidis, q. s. ut fiat pilula, tertia quaque hora sumenda. Superbibendo haustum sequentem.
Infusi Rosæ, ℥ xj.; Acid. Sulph. dil. m. xij.; Syrupi, ℥ i. M.
Vini rubri, ℥ iij. 6tis horis.

3d.—No sickness since yesterday. Medicines retained. Tongue moist and clean; some appearance of aphthæ. Two evacuations, —the first tinged with blood, and the second chiefly consisting of it. Abdomen flabby, soft, and *without the slightest tenderness;* pulse 140; a spot on the nates threatens to slough.

Injeciatur Enema, ex aqua frigidissima si redierint dejectiones sanguineæ et rep. si opus sit. Rep. medicamenta.

6th.—Continued to sink gradually, and died at three o'clock this morning. The abdomen became tympanitic, but *at no period was there any tenderness on pressure.* No blood observed in the stools since the 3d. The petechiæ did not increase; the medicines were retained; and she understood the questions put to her at the visit yesterday.

*Examination of the body, ten hours after death.—Abdomen:* On opening the cavity of the abdomen, the intestines, particularly the colon, were observed to be distended with flatus; the peritoneal covering of the bowels was of a light lilac colour, and the mesenteric glands appeared turgid with ecchymosed blood. The mucous membrane of the ilium and colon was partially elevated, as if from blood extravasated under it. In the midst of these ecchymosed parts were observed the mucous glands projecting, and in some instances ulcerated. In some parts there was ecchymosis without any enlarged glands perceptible, either to the eye or to the finger: in others, there were enlarged glands studded over the membrane, with little or no ecchymosis; and yet in others there were chancre-like ulcerations, as large as a sixpence. There was no increased secretion of mucus, and the fæculent matters which remained in the intestines were of a rhubarb colour. The liver was rather pale, but otherwise healthy.

*Thorax:* A portion of the diaphragm, and corresponding part of the cardiac extremity of the stomach, were eroded, by which a quantity of fluid had escaped into the thorax. There were three apertures,—one as large as three-fourths of the palm of the hand, and two others about the size of sixpence: the edges of all were quite sharp and clean, without surrounding thickening of the mucous membrane. They were considered to have occurred after death. There were about two ounces of fluid in the pericardium, and some old adhesions of the lungs.

*Head:* About two ounces of serum were effused between the membranes of the brain. There was no accumulation in the ventricles. The pia mater was rather injected, and the substance of the brain exhibited several red spots, on cutting it transversely.

II. *Follicular Ulcerations, with slight tenderness of the abdomen at the time of admission, but not experienced again until after a fortnight.*

Charles Sedgewick, aged twenty-two; admitted March 1, 1826.

Felt some indisposition three weeks ago, and in a week after was attacked with distinct rigors, and other precursory symptoms of fever. At present his tongue is excessively dry and dark, resembling old mahogany; he has tenderness of the hypogastrium, and tension of the abdomen; the bowels are stated to have been much relaxed during the whole attack; his pulse is frequent and fluttering; the skin is cool. After he had been an hour in bed, the skin became hot and dry, and the pulse increased in strength.

Calomel, Pulv. Jacobi (veri), āā gr. iv. fiant pil. duæ, statim sumendæ; post horas tres sumatur Haustus Sennæ.

Repetatur pilula una, hora somni et cras mane.

Haustus Salin. c. Liq. Antimon. Tartar. ℥ss. quater die sumendus.

March 2d.—Only one stool. Tongue becoming moist, but with a very thick fur adhering in the centre; pulse ninety-two to ninety-six, of sufficient strength; skin warm and dry. No delirium.

Calomel, gr. v.; Pulv. Jalap. gr. xij. M. fiant pil. iij. quam primum sumend. Haustus Sennæ cras mane nisi interim plene dejic. alvus.

Repet. Haustus Salin. c. Liq. Antimon. Tartar.

3d.—Bowels very freely opened: motions liquid and dark. In other respects as yesterday.

Repetatur Haustus Salin. Omittantur alia.

4th.—Bowels very open: motions liquid, natural. Skin cool; pulse ninety-six to an hundred, soft.

Sulphat. Quinæ, gr. ij. in formâ pil. 4tâ q. hora; superbibendo haustum seq.

Infusi Rosæ, ℥ xj.; Acid. Sulph. dilut. m. v.; Syrupi Aurantii, ℥ i.; Tr. Opii, m. ij.

8th.—Tongue moist and cleaning, but very slowly; skin cool; ulse eighty-eight to ninety, small and soft.

Vini Hispanici, ℥ iij. quotidie.

9th.—Tongue has again assumed an unfavourable appearance. Abdomen tense; bowels have not acted since yesterday; pulse soft; skin cool.

Calomel, gr. iv.; Pulv. Rhei, Scammoniæ, āā gr. viij. fiat pulvis statim e melle sumendus, et cras mane si opus sit repetendus.

Mist. Camphoræ, ℥ xj.; Carb. Ammon. gr. v.; Confect. Arom. ℈i.; Syrupi Aurantii, ℥ i. fiat haustus quater in die sumendus. Omitt. alia.

11th.—The purgative medicines were repeated yesterday. Bowels freely open during the last two days. Tongue seems inclined to throw off its crust. Abdomen less tumid and hard; still is not sufficiently supple. Pulse ninety-two, soft.

Ol. Ricini, ℥ iij. c. Mucilag. Acaciæ et Aq. Cinnam. statim et omni mane.

Omitt. Haustus, et resumant. Pil. Quinæ. Habeat Vini albi, ℥ iij. quotidie.

15th.—Bowels have been open three or four times in the day since last report: stools liquid, but otherwise natural. Pressure on the abdomen has been acknowledged to *give pain, for the first time since the day of admission:* the abdomen is no longer tense. Tongue very much loaded; skin hot, harsh, and dry; pulse 100 to 104, not so soft as heretofore. Says he has no uneasiness whatever, except the sense of weakness.

Hydrar. Submuriat., Pulv. Jacobi (veri), āā gr. ij. in formâ pilulæ, 6tis horis.

Repet. Mistura Camphoræ, &c. Omitt. alia.

23d.—Medicines ordered on 15th have been continued. The bowels have been kept freely open, and the tongue is very much improved. The abdomen has *been rather tense*, but *not tender on pressure.* The pulse has become more expanded, varying from ninety to an hundred; and he has, upon the whole, appeared to improve, though very slowly. On the 21st, he complained of some sore throat, and the fauces were observed to be more red and inflamed than the slight mercurial action on the gums was capable of explaining. To-day, the glands under the angle of the jaw are swollen on the left side, and very painful.

Cataplasma Lini faucibus externis, quater in die renovandum.

Resum. Pil. Sulphat. Quinæ, gr. ij. 6tis horis. Superbibendo Haustum sequentem.

Acid. Sulph. dil. m. x.; Sulphat. Magnesiæ, ʒ ss.; Syrupi Rosæ. ʒ i.; Infusi Rosæ, ℥ xj.

Repetatur Vinum. Omitt. alia.

26th.—Bowels freely open; abdomen is now supple. Tongue moist, but with some adhering fur; pulse very small and frequent; skin cool.

April 1st.—Continued to linger till this afternoon, when he died, in the eighth week of the fever. Little of importance took place in the train of symptoms since last report. The strength continued gradually declining, and the quantity of the stimulants was proportionally increased. The bowels continued to act once or twice daily, the stools presenting nothing remarkable in their appearance; the abdomen having latterly become sunk and hollow. Neither was there any pain on pressure till two days ago, at which time he complained equally when the pressure was applied to other parts,—as over the ribs. The pain and tenderness about the throat continued, and some muco-purulent matter was spit up. On the 30th, he complained for *the first time* of pain in the left side of the thorax, which continued till his death.

*Examination of the body, about twenty-four hours after death.*—*Head:* Brain sound; about two ounces of serosity in the ventricles.

*Throat and Chest:* Abscess at the angle of the jaw, involving the neighbouring parts by burrowing into the surrounding cellular tissue, and even bursting into the larynx, a little above the left sacculus laryngi. Extensive adhesions of left lung; recent coagulable lymph. From six to eight ounces of fluid in the pericardium.

*Abdomen:* A considerable number of follicular ulcers, beginning in the small intestines, becoming more numerous and extensive towards the ileo-cœcal valve, and gradually disappearing in the colon.

III. *Case of Follicular Ulceration, existing during the period of Convalescence from Idiopathic Fever, and exciting fatal Peritonitis, although none of the ulcers had perforated the Peritoneum.*

On Friday, September 23d, ten o'clock A.M. 1825, I was sent for to see a maid-servant, aged thirty, in a private family.

I learnt that she had been long ailing (for six or seven weeks), with symptoms of fever, for which venesection and the appropriate treatment had been employed; that the fever had gradually assumed a decidedly remittent character, but had been so completely removed by Sulph. Quinæ, that, on Sunday last (the 18th instant), she had left her sick room, with the view of exercising herself in the house, though not yet sufficiently recovered to resume her ordinary work. On Monday night she had a distinct rigor. On

Tuesday morning was seen by a medical gentleman, who purged her with calomel and rhubarb. She did not then complain (or even on Wednesday morning) of any pain or tenderness of the body, but in the afternoon experienced an attack of pain in the epigastrium, and vomiting, for which she was immediately cupped, and had a large blister applied to the abdomen.

On the Friday morning, seven o'clock A.M. she had been bled almost to syncope for symptoms of unequivocal peritonitis. When I saw her, at ten o'clock A.M., there was exquisite tenderness about the right iliac region and hypogastrium, with dysuria, "deadly sickness," incessant vomiting, frequent and sharp wiry pulse, and dry blood-red tongue. The bowels had lately acted slightly; and she was perspiring profusely, having lately come out of a warm bath.

Venesection was immediately repeated, and again in the afternoon, followed by leeches in the evening. Venesection was repeated at two o'clock P.M. of Saturday. The bleeding was on all these occasions pushed almost to syncope; the blood was highly cupped and buffy. Only a temporary mitigation of the sufferings was obtained after each venesection. No purgatives, only enemata, were employed.

During the Saturday she retained two calomel-powders, each containing ten grains.

At eleven o'clock P.M. she was incessantly complaining both of the pain of the blister and also of the internal pain, which she compared, in her own emphatic language, "to a cauldron of pitch and sulphur burning within her." Skin was quite cold and clammy; pulse very frequent and feeble; and latterly she had occasionally been delirious for a few seconds, but soon recovered her intellects.

Sunday, eight o'clock A.M. she died, having retained her faculties quite clear until seven o'clock, when the last visit was paid by her medical attendant.

*Examination of the body, two hours and a quarter after death.*—*Lungs* beautifully healthy.

*Abdomen:* The peritoneal coat of the intestines had lost its proper degree of transparency, but there were no other marks of peritoneal inflammation; no effusion of coagulable lymph or serum in the cavity of the abdomen. Liver healthy. Gall-bladder distended with good bile. A quantity of bile was also observed in the stomach, of a green colour; but in the duodenum, and throughout the whole track of the intestines, the bile was found in large quantity, and of a perfectly good colour. The mucous membrane of the stomach was slightly inflamed, a pink or salmon colour pervading it universally—even the rugæ; while that of the duodenum and small intestines was perfectly healthy: that, however, of the caput coli, colon, and rectum, was inflamed. Low in the ascending arch of the colon, about an inch from the ileo-cœcal valve, were discovered two small, but deep, follicular ulcers; one of them half an inch long, scarcely onetenth of an inch wide: its base was not distinctly visible, being obscured by some protruding little masses of obstructed and enlarged follicles. In many other parts of the colon, though there was no visible ulceration, still there might be detected similar little follicles, enlarged and slightly elevating the mucous membrane. The mesenteric glands were very much enlarged and reddened: on cutting into one of them, it resembled in consistence the pulp of a ~~white-heart~~ cherry, and exhibited rather a deeper tint.

IV. *Case of Follicular Ulcerations, one of which penetrated through the peritoneal coat, and excited universal Peritonitis.*

Sarah Rawdon, ætat. twenty-three; admitted May 24, 1826.

Left service three days ago, and is stated by her attendant to have been noisy and delirious at night ever since: at present is perfectly rational. Says she has had a cough for three or four weeks, and became seriously ill only on this day se'nnight, with rigors and the usual premonitory symptoms of fever. Some days before her admission, a dose of jalap was administered, since which time the bowels have been much relaxed. The abdomen is very tense and tender. Had severe purging last night, with much pain in the belly, for which she took some brandy, and she says with great relief. Tongue dry in the centre, moist and clammy at the edges; pulse 112, soft; respiration short and hurried; eye natural; little or no headache.

Hydrar. Submur. gr. v.; Scammon. gr. xij. M. fiat pulvis statim sumendus.

Hydrar. Submur., Pulv. Jacobi (veri), āā gr. ij.; Mucil. Acaciæ, q. s. ut fiat pilula tertiis horis sumenda.

Haustus Ammoniæ acet. quater in die.—Lotio spirituosa fronti, collo, et branchiis, si incalescat cutis.

May 25th.—Has had only one evacuation, and that almost immediately after admission, (not kept). Abdomen distended, occasionally very painful; the pain moveable, and affecting different regions at different times: pressure excites pain, but so it does if applied over the ribs. Any movement gives pain. She is lying on the right side. Pulse 120, soft, small; tongue dry, brown; skin harsh, not hot. Was quiet during the night.

Ol. Ricini, ʒ iij.; Mucilag. Acaciæ, q. s.; Aq. Cinnam. ʒ vj. fiat haustus statim sumendus; et repetatur 2dis horis donec bis terve dejecerit alvus.

Mist. Camphoræ, ℥ i.; Liq. Ammoniæ acet. ʒ iij.; Spiritus Ætheris Nit., Syrupi Aurantii, āā ʒ ss. 4tis horis.—Repetantur pilulæ.

26th.—Had marked rigors and vomiting in the course of yesterday, followed by cold clammy perspiration, and died in the evening.

*Examination of the body,* (26th.)—*Abdomen:* A large quantity of discoloured serous fluid, with universal peritonitis. An aperture, as large as a split pea, in the ileum. On examining the inner surface of the bowel, several follicular ulcers were found, but none of them had penetrated farther than the mucous and muscular coats, with the exception of the one above mentioned. That this ulceration had proceeded from within outwards, —i. e. that it had commenced in follicular enlargement and ulceration, producing erosion of the subjacent peritoneal coat, was obviously shown by the different size and appearance of the ulcer, as viewed from the side of the peritoneum or of the mucous membrane. The perforation of the peritoneum did not exceed the size of a split pea, while the erosion of the muscular and mucous coats extended over a space not less than a shilling; its margins being raised and jagged, and presenting an appearance exactly resembling that hereafter described as the effect of follicular ulceration. The mucous membrane of the stomach was highly vascular.

*Thorax:* Lungs partially hepatised.

*Head:* A small quantity of fluid between the arachnoid and pia mater, and also in the lateral ventricles.

V. *Follicular Ulceration reaching the Peritoneum, and exciting adhesive inflammation there, by which the part became agglutinated to the descending flap of the omentum, and the continuity of the intestinal canal was preserved.*

Eliza Parton, ætat. twenty, single; admitted March 16th. Abdomen was then tense, but not tender. She had been attacked with fever eleven days before. Died March 30th.

*Examination of the body*, March 31st.—*Abdomen:* In the lower part of the ileum, there were several follicular ulcers, one as large as half-a-crown, occupying the confines and part of the ileo-cœcal valve: it had nearly penetrated through all the coats, but fortunately the inflammation excited in the peritoneal coat had prevented its erosion, and had caused its adhesion to the descending flap of the omentum. In the ascending arch of the colon, there were several follicular ulcers, of the size and appearance of small-pox pustules: in its descending arch there were numerous black specks, indicating the obstructed orifices of the slightly-enlarged mucous follicles, like acne punctata, and clearly explaining the first step in the progress of disorganisation by follicular ulceration.

The mesenteric glands, corresponding to these follicular ulcers, were much enlarged and reddened.

*Thorax:* There was no increased vascularity in the bronchial membrane, or in that of the trachea; and little or none in the substance of the lungs.

*Head:* The lateral ventricles of the brain contained a very small quantity of fluid; the plexus choroides was pallid; the substance of the brain natural.

N. B.—This girl never had the slightest tenderness of the abdomen: indeed, it became soft and supple after the operation of the first purgative. Neither was there any blood in the evacuations; though there was visible a slow exhalation of blood from the gums and nose, forming a considerable accumulation of black sordes. The tongue also became aphthous, fissured, dry, and black. There was also slight subsultus tendinum, but no movement resembling that of catching flies. There was night-delirium; but in the day she recovered her faculties, and retained them even on the day of her death.

### VI. *Follicular Ulceration, fatal within eight days.*

Mary Hawkins, ætat. twenty; admitted November 26th, with fever of a week's standing, and the following symptoms:—She was in a state of high delirium; the temporal arteries throbbing violently, and the pupils much dilated; face pallid; pulse very frequent, but could not be easily counted, or its precise character ascertained, on account of the frequent convulsive startings of the muscles of the arms; skin warm and moist; tongue dry in centre; abdomen full, but not tense. Could not bear pressure on the right iliac region without expression of extreme uneasiness; the other parts of the abdomen were quite free from tenderness on firm pressure.

The medical gentleman, who had attended her previously to her admission into the hospital, stated that she had been ill about a week, with fever and suppression of the catamenia, from cold caught after dancing. There had been night delirium, some stupor and deafness by day, with torpid bowels. He had purged her freely, and given calomel, with antimonial powder, 4tis horis; and had taken thirty ounces of blood by cupping, the day before her admission.

The following means were employed in the hospital:

Applic. Hirudines xvj. fronti et temporibus statim. Glacies capiti raso.
Pulv. Jalapæ c. Calomel, gr. xxv. statim.
Calomelanos, Pulv. Antimon. āā gr. v. horâ somni.

She became tranquil after the application of the leeches and ice to the head; the pupils of the eyes resumed their natural state, and the subsultus tendinum was much diminished. The bowels acted once scantily. She died in the night, about twelve hours after her admission.

*Sectio cadaveris*, (Nov. 27th.)—*Head:* Brain natural, excepting for the presence of about three drachms of serum in the lateral ventricles, and also some small quantity between the membranes.

*Thorax:* The mucous membrane of the trachea and bronchia slightly injected with red vessels.

*Abdomen:* About the ileo-cœcal portion of the intestines, there were numerous enlarged follicles; some merely protruding the mucous membrane, others producing ulceration in it, with little or no redness. The mesenteric glands corresponding to these ulcers were considerably enlarged.

### VII. *Follicular Ulceration, the abdomen being supple, and not tender.*

Charles Eynon, ætat. twenty-five; admitted November 22d. A week before his admission, he had been attacked with shivering, followed by fever and night delirium. At present there is slight bleeding from the gums, with accumulation of black sordes about the teeth; moist tongue; considerable deafness, but, when spoken to loudly, he attempts to protrude his tongue; pupils rather dilated, and sluggish in contracting. He does not acknowledge any pain upon pressure of abdomen, which is soft and flat. Bowels are relaxed, but no blood has been observed in the stools.

Died November 28th.

*Sectio cadaveris*, (November 29th.)—There was found extensive ulceration of the glandulæ aggregatæ, although pressure on the abdomen, tried daily, never excited any pain or complaint. Perhaps, in this case, the insensibility to pain may be attributable to the impaired functions of the cerebral system.

VIII. *Follicular Ulceration, with the appearance of black puncta at the orifices of the follicles.*

William Gibbs, ætat. twenty-five; admitted November 21st, having been ill with fever some days. Obiit December 5th.

*Sectio cadaveris*, (December 6th, 1826,) displayed follicular obstruction, with black puncta at the orifices, like acne punctata, and follicular ulcerations in the ileo-cœcal portion of the intestines.

From the time of his admission until Saturday, November 26th, he appeared torpid and stupid; the pupils contracting sluggishly, the eyes having a lack-lustre appearance, and no satisfactory answers being given by him to the questions asked respecting his health: the tongue, however, was quite moist, and almost clean; the pulse about ninety, and soft; skin soft, and not particularly hot; abdomen fullish, and rather tender. He was delirious at night, and made frequent attempts to get out of bed. On the night of Friday, November 25th, very active diarrhœa came on; and, during the visit on the following day, I observed him in full possession of his faculties, which he retained until the moment of his death; the diarrhœa still continuing, though not so actively, and being accompanied with some tenderness of the abdomen. The immediate cause of death appeared to be extensive mortification of the left leg, spreading from a spot where he acknowledged to have received a blow some considerable time before.

IX. *Follicular Ulcerations distinctly visible upon exposure of the cavity of the abdomen, so perfectly had the peritoneal and muscular coats retained their natural degree of transparency.*

Elizabeth Lawrence, æt. twenty, was admitted December 14th; had been then ailing eleven days with fever; died January 4th, 1826.

At the time of her admission, there was tenderness on pressure of the abdomen, but no swelling or tension of it; specks of blood, too, had been observed in the evacuations. She made no complaint of headache, but of a noise and ringing in her ears. The intellects, however, were then quite clear, and remained so until night-delirium came on about a week before her death; but even then during the day she recovered the full possession of her faculties. The eyes retained their natural appearance, until within two days of her death, when the pupils became rather dilated. She was much distressed, too, by a harrassing cough.

*Sectio cadaveris,* (January 6th.)—*Head:* The brain was healthy, except that there was about half an ounce of clear serum in the lateral ventricles.

*Thorax:* The cavity of the pericardium contained about three ounces of clear serum. The mucous membrane of the larynx, trachea, and bronchia, was highly injected and inflamed; and the greater part of the lungs was so completely hepatised as to sink in water, only a very small portion retaining the property of crepitation.

*Abdomen:* The peritoneum was quite healthy, and so transparent as to allow us, immediately upon opening the cavity of the abdomen, to see numerous follicular ulcers, which began about the ileum, but were most frequent about the ileo-cœcal valve.

X. *Follicular Ulcerations, some of which were apparently healing.*

Nathaniel Shoultee, a German, æt. thirty-two; admitted Monday, July 3d, 1826. Lives in Pimlico. Shows his tongue when desired; seems to understand the questions put to him by his German companions, but fails in answering from weakness. He is now lying quiet. Present symptoms are—small, soft pulse, 144; sluggish pupils; conjunctiva natural, and no flushing of the face, though the forehead is hot; tongue moist, but furred; gums and membrane of cheek slightly affected by mercury; abdomen feels natural, but, when it was first pressed, he started with the expression of pain; but so also he did when pressure was made above the ribs, and soon afterwards he bore firm pressure on the abdomen without evincing any pain. Skin cool and clammy; respiration much hurried, and executed by the diaphragm alone, without any elevation of ribs by intercostal muscles.

His friend states that, on Wednesday, the 14th of June, he was attacked with fever, having been previously well; but cannot give any further particulars, excepting that, for the last three or four days, his evacuations have been passed involuntarily.

Radatur Capillitium, et applic. Lotio frigida.

Applic. Emplastrum Lyttæ nuchæ.

Mist. Camphoræ, ʒxj.; Confect. Aromat. ℈i.; Carbon. Ammoniæ, gr. v.; Syrupi Aurantii, ʒi. M. fiat 3tiis horis.

Vini albi, ℥iij. ex Aquæ, repetend. prout deficiant vires.

Hydrarg. c. Cretâ, gr. v. 4tis horis.

Olei Ricini, ʒij.; Mucil. Acaciæ, q. s.; Aquæ Cinnam. ʒvj. M. fiat haustus, cras mane sumendum.

Obiit early on the morning of the 4th instant.

*Sectio cadaveris, eight hours after death.*—*Brain:* Some small quantity of fluid between the arachnoid and pia mater, and great congestion of the veins running between the convolutions on the convexities of the hemispheres. Substance of the brain highly vascular, as was the plexus choroides. About two ounces of clear serum in the lateral ventricles, and also a large quantity in the theca vertebralis. There was no effusion of coagulable lymph about the pons varolii, or elsewhere.

*Thorax:* Lungs were in a state of congestion; but the mucous membrane of the larynx and trachea was only very slightly injected with red vessels.

*Abdomen:* The peritoneum retained its natural transparency, excepting in some nebulous patches of the ileum, found, upon opening the cavity of the intestines, to cover reticulated ulcers, presenting the appearance supposed to indicate the progressive healing of follicular ulcers.* But, besides these eroded surfaces, the mucous follicles about the ileo-cœcal portion were much developed, with little or no surrounding inflammation, and passing into ulceration at their previously-obstructed orifices. The obstructed condition of these orifices was clearly visible in other follicles along the course of the colon, seen at an earlier stage of their enlargement, and was indicated by the black puncta, analogous to acne punctata.

The mesenteric glands were slightly enlarged, but very much reddened; one of them, when cut into, very closely resembled, in consistence and colour, the pulp of a red cherry.

### PATHOLOGICAL OBSERVATIONS.

My first object, in detailing the above cases, has been to exhibit the origin and progress of the disorganising process, by which follicular ulceration is effected in the mucous membrane of the intestines, during the course of idiopathic fever.

A reference to the above post-mortem examinations will show that the disease seems at first to be limited to the mucous follicles, affecting both the glandulæ aggregatæ and solitariæ, more especially the former, where distributed over the ileo-cœcal portion of the intestines;—that their orifices may be seen plugged up with a dark and morbidly inspissated secretion;—that this secretion continuing, and being pent up within the follicle, in consequence of the obstruction of its orifice, tumefaction of the gland necessarily ensues, as in the sebaceous follicles in acne punctata; that these mucous glands may be seen in various stages of enlargement,—at first only slightly elevating the mucous membrane, and, by their protrusion of it, presenting an uneven granulated surface, like that of dried seal-skin;—that, in most instances

* Vide Pathological Observations below.

the incipient tumefaction of the follicles, and in many instances their ulceration, takes place without any increased vascularity of the neighbouring surface;—that the disorganising process, by affecting many of the contiguous mucous glands, forms ulcers of the size of sixpence or a shilling, or even larger in some instances, without any increased vascularity, but more generally with slight redness and considerable thickening of the mucous membrane immediately surrounding the margin of the ulcer;—that the margins of the ulcer frequently appear, at least in the early stages, puckered, jagged, or fringed;—that the base of such ulcers often exhibits a rough irregular surface, with little projecting masses of enlarged and sloughing follicles, or presents a sloughy ash-coloured appearance, resembling that of chancre, or displays a dirty-yellow hue, from the bilious-coloured feculent matter adhering to it;—that the ulceration seems to commence sometimes at the apex of the tumefied gland, sometimes in the membrane protruded, and thus forced into ulceration by it;—that the work of disorganisation seems to be effected partly by the ulcerative, partly by the sloughing process;—that each individual follicle gradually sloughs off, in proportion as its neighbouring membrane is eroded by ulceration;—that this erosion extends gradually downwards, from the mucous to the muscular and peritoneal coats, sometimes, though rarely, perforating the latter, and then rapidly exciting extensive peritonitis, by the escape of foreign matter into the cavity of the abdomen;—that this perforation of the peritoneal coat is sometimes prevented, by the effusion of lymph from its surface, external to the ulcer, causing agglutination of it to some of the neighbouring convolutions of the intestines, or to the omentum;*—that the mesenteric glands, communicating with such ulcers, are generally found increased in size and vascularity; and sometimes, when cut into, resembling, in colour and consistence, the pulp of a common ~~Morello cherry, or even that of a~~ red cherry.

The symptoms, too, attending the recovery of some of the cases, hereafter to be described, seem to warrant the belief that such ulcers had existed, but had subsequently healed.

An additional, and I think conclusive, proof of the curability of intestinal ulcers, (already asserted, and very ably maintained, by Dr. P. Latham,) was afforded to me by the appearances discovered in the intestines of a sailor (George Gratlin), who, about fourteen months previously to his death, had experienced, while serving off the coast of Africa,

* Vide Sectio cadaveris, Eliza Parton.

a severe attack of dysentery. From this he slowly recovered sufficiently to return to his duty, though subject ever afterwards to occasional irregularities of the bowels, and to an obscure sense of pain when forcible pressure was applied to the abdomen. He died December 22d, 1825, in St. George's Hospital, while under my treatment for a disease quite unconnected with his former dysenteric attack, and about fourteen months subsequently to it.

Upon examination of the intestines, the mucous membrane, from two or three inches above the ileo-cœcal valve along the whole course of the colon and rectum, appeared spangled with circular or irregularly-oval scars of former ulcers. Some of them, in which the process of reparation had not yet been completed, exhibited the following appearances:—Along the base and margin of these healing ulcers, there was distinctly visible a thin film of yellowish-white coagulable lymph, which seemed to be gradually proceeding to fill up the ulcerated cavity. In many instances it had not yet reached the level of the adjoining surface; in others, it had: in the latter the regeneration of the surface seemed complete, the lymph having become so intimately blended with the neighbouring membrane as to be scarcely distinguishable, except by the glistening and spangled appearance above mentioned. In the former the process of reparation seemed to be gradually advancing: in these, the neighbouring edge of the mucous membrane (which had probably been previously thickened and fringed,) appeared to be gradually subsiding, contracting and bevelling itself down so as to favour the progress of the cicatrisation. Where the breach of surface had been completely repaired, there was no increased vascularity; and only a very faint appearance of it in the ulcers undergoing the healing process; the degree of redness seeming to vary according to the exigencies of each individual ulcer, being more or less in proportion as more or less lymph was required for the filling-up of the ulcer, and gradually becoming evanescent, as the continuity of the surface was re-established.

The healing of these ulcers appeared to me to be effected, not, as in external ulcers, by any visible growth of granulations, but by a process analogous to that which is displayed in the reparation of ulcers of the cornea by the deposition of lymph.

In the healing of follicular ulcers, (though, to the eye of the observer, there may be an apparent regeneration of the mucous membrane, completely obliterating the former breach of surface,) still, though the continuity of the membrane is re-established, yet the parts so healed over must remain permanently unprovided with mucous follicles; for, previously to the commencement of the healing part of the process, it seems to be essentially necessary that the ulcerating follicles should have sloughed off.

This restorative power, too, seems to be limited merely to the re-establishment of the continuity of the exhalant surface, and does not extend to the regeneration either of the follicles, as just stated, or of any the smallest portion of the valvulæ conniventes, which may have been eroded. I have had opportunities of distinctly tracing such ulcers, which have apparently been undergoing the healing process, merely by the contraction of their margins and the effusion of lymph, without any attempt at the reproduction of the eroded part of the valvula connivens.

In most of the cases of follicular ulcers of the intestines in idiopathic fever, the seat of the ulcers will be indicated, upon opening the cavity of the abdomen, by some deviation from the natural transparency of the peritoneum, either by small opaque patches of silvery whiteness, or by apparently attenuated spots, occurring in discoloured portions of the intestines. In one case, (vide Elizabeth Lawson,) the peritoneum retained its natural transparency so perfectly as to allow me to distinguish most accurately the seat and extent of the different follicular ulcers, before the cavity of the intestines was opened. But it not unfrequently happens that there are no peculiar appearances of the peritoneum, to indicate the existence of these follicular ulcers; and hence they have frequently been overlooked.

To those who have not the opportunity of testing the accuracy of these statements, by frequent inspection of the intestines in fatal cases of idiopathic fever, and still are desirous of becoming acquainted with the manner and progress of the follicular ulceration above described, it may perhaps be satisfactory to learn that, frequently after death produced by phthisis tubercularis, attended with colliquative diarrhœa, a similar, though slower, process of disorganisation may be detected, first attacking the mucous glands, more particularly those of the ileum; and then eroding the muscular, and sometimes, though very rarely, even the peritoneal coat.

That a greater resistance is made by the peritoneal than by the other coats, to the progress of this ulcerative action, is rendered evident in fatal cases of phthisis tubercularis, attended with follicular ulcerations of the intestines; for the ulcers are often numerous, and may be seen in various stages of their progress,—many of them penetrating through the mucous and muscular coats, so as to expose the interior of the peritoneal coat for the extent sometimes of an inch in diameter, without however perforating it.

Besides this mode of follicular ulceration, there are two other morbid appearances, which I have occasionally detected in the mucous membrane of the intestines of those who have died with protracted fever, at a period when abdominal derangement had entirely subsided, or was not remarked as a prominent feature of the disease.

One of these morbid appearances may be described as consisting of light buff-coloured patches of various forms and sizes, without elevated borders, but with a well-defined outline, marked by the sudden difference of colour between them and the adjoining surface. The enclosed area is traversed by numerous thin seams, or septa, forming by their interlacement minute cellules, the base of which appears to be either the denuded peritoneum, or a thin nearly-transparent film of lymph deposited upon that membrane. The delineation by words of such alterations of structure must always be imperfect, but perhaps the imagination of the reader may be enabled to picture to itself the appearance meant to be described, if it be stated that it may be not inaptly compared to the texture of the wing of a dragon-fly, or the skeleton of a decayed leaf. .

The other morbid appearance above alluded to, so nearly resembles the one just described, that the only mark of difference consists in the slight elevation of the borders and septa by the deposition of lymph : consequently, the coats of the intestine at these spots, instead of being, as in the former case, attenuated, are slightly thickened ; and the seat of the altered structure may be recognised, before the cavity of the intestine is opened, by the silvery opacity of the peritoneum, which, however, while passing over it, exhibits no irregularity, whether of puckering inwards or projection outwards, but retains its uniformly level and smooth surface.

These two appearances seem to me to display the progressive stages of the healing process, after the separation of the follicles, to the ultimate regeneration of the membranous surface.

In the progress towards follicular ulceration, though a cluster of these glands may have become simultaneously enlarged, and have thus, by their pressure upwards, caused erosion of their investing membrane, still the latter is not always wholly eroded, but only partially around each follicle, to the extent of allowing the separation of the follicle through the perforation, while the still surviving filaments of the membrane form the septa enclosing the cellules left after the escape of the follicles: It is from these septa, as well as from the base of each cellule, that lymph is effused, to repair the breach of surface. This deposition at first produces a slight elevation and opacity about the septa and margins of the ulcers, but it is gradually converted into the nature of the neighbouring membrane, assuming at the same time the same level and the same degree of transparency.

Besides these follicular ulcerations, I have occasionally observed the ecchymosed spots and ulcers, so admirably described by Dr. P. Latham; and recently, since writing the above observation, I have read, in the Number of the Medico-Chirurgical Review of July, 1826, some extracts from a paper by Dr. BRETONNEAU, of Tours, entitled *Dothinenteritis*. I am very happy to add my evidence in favour of the general accuracy of his description of these alterations of structure, though I readily confess myself unable to depict, as he has attempted to do, the daily changes in the appearance of these follicular ulcers of the intestines, with a precision equalling that with which the daily progress of variolous pustules may be delineated.

In conclusion, I beg to observe, that the above remarks are directed exclusively to the elucidation of the pathology of the follicular ulceration of the mucous membrane of the intestines in idiopathic fever: in their origin and progress they are as different, as in their termination, from that superficial abrasion, which has gained the distinctive appellation of *abrasive ulceration* of the mucous membrane of the intestines.

I suspect, however, that these follicular ulcerations in the intestines are of much more frequent occurrence than is generally imagined. During the sectio cadaveris of a young girl, who died at St. George's Hospital, in August 1825, four or five days after a very extensive burn, I found numerous follicles, enlarged and protruding the mucous membrane, all along the course of the ilium and colon, some of them just passing into a state of ulceration at their most prominent part. These appearances not only remain still vividly impressed upon my recollection, but are recorded in my notes, written immediately after the dissection.

In the next Number, an attempt will be made to ascertain the period of idiopathic fever, at which this follicular ulceration of the intestines usually appears; and a plan of treatment will be proposed, founded upon the above pathological views, and illustrated by the successful termination of cases, notwithstanding the occurrence of symptoms indicating, or supposed to indicate, the existence of these follicular ulcerations.

At present, I would only beg permission to state, that these pathological views afford an additional and powerful argument for active and persevering purgation by effective doses of calomel, combined with any of the usual auxiliary purgatives, in the early stage of fever. Its power of preventing follicular ulceration, seems readily explicable on the following principles:—The preceding investigations appear to have established that the first step towards follicular ulceration is obstruction of the orifice of the gland by its own morbidly-inspissated secretion; the next step being distention, from the continuance and accumulation within it of this secretion, and subsequently ulceration. If, then, at this period effective purgatives be employed, they will clear out the obstructed orifices, and thus anticipate the distention and subsequent ulceration of the follicles.

---

### RHEUMATISM.

ERRATA.—In pages 103 and 109, for "white-heart" and "Morella" cherry, read "common red cherry."

